# The Adolescent HIV Communication Belief Scale: Preliminary Reliability and Validity

**DOI:** 10.1007/s10826-018-1075-7

**Published:** 2018-03-27

**Authors:** Michael Evangeli

**Affiliations:** Department of Psychology, Royal Holloway University of London, Egham, UK

**Keywords:** HIV, Communication, Adolescent, Questionnaire, Reliability

## Abstract

Globally, there are nearly 2 million HIV positive children, many of whom are adolescents. The majority have perinatally acquired HIV. A key challenge for this population is communicating about HIV to meet emotional and practical needs. Despite evidence of its benefits, HIV communication in adolescents with HIV is rare. To enhance HIV communication, individuals’ beliefs may need to be taken into account. There is no measure of beliefs about HIV communication for adolescents living with HIV. A seven-item measure of HIV communication beliefs was developed and administered to 66 adolescents with HIV in the UK (39 female; aged 12–16 years). Data were explored using principal component analysis. Preliminary criterion-related validity was assessed by examining relationships between the measure and communication occurrence, frequency and intention. Preliminary construct validity was assessed by examining relationships between the measure and HIV stigma, HIV disclosure cognition and affect, quality of life and self-perception. Two factors were revealed: communication self-efficacy and normative beliefs; and communication attitudes. The full scale and its subscales were internally consistent. The total score showed statistically significant positive relationships with HIV communication intention, HIV disclosure cognitions and affect, and HIV stigma but not with other variables. Preliminary evidence of the measure’s good psychometric properties suggests it may be helpful in outlining relationships between HIV communication beliefs and other constructs. It may also be useful in testing interventions that aim to enhance HIV communication in this population. Further work needs to be done to establish the scale’s psychometric properties.

## Introduction

An estimated 1.8 million children were living with HIV in 2015, many of whom are adolescents, mostly in Sub-Saharan Africa (UNAIDS 2016). The majority have perinatally acquired HIV (PAH). With advances in antiretroviral therapy (ART), children born with HIV can have comparable life expectancy to HIV-negative children (Wada et al. 2014). Adolescents with HIV do, however, face a number of challenges. These include adjusting to being told that one is HIV-positive (paediatric disclosure or naming), managing long term ART adherence often with histories of suboptimal regimens (Sohn and Hazra 2013), and anxiety about sharing one’s status (onward HIV disclosure), particularly in the context of the onset of intimate relationships. In addition, many have experienced multiple caretaking transitions with parental illness or death, hospitalisations, missed school and social opportunities, and pain (Mellins and Malee 2013). Possibly as a result, there are higher levels of emotional and behavioural problems, including psychiatric disorders, in young people with PAH compared to young people unaffected by HIV (Mellins and Malee 2013).

Communicating about HIV with family, friends and partners may help in managing HIV-related challenges. Effective communication may allow adolescents with HIV to express their needs and concerns and provides opportunities to share information and receive support from others. Communication about HIV may create feelings of closeness in key relationships and help in the management of HIV stigma (Proulx-Boucher et al. 2017). In other populations with HIV, greater health-protective sexual communication (including communication about HIV) has been associated with less sexual risk behaviour (Serovich et al. 2017). Some psychosocial interventions involving adolescents with HIV aim to enhance communication (not specifically HIV-related) between adolescents and caregivers. These interventions have been effective in enhancing adolescent emotional wellbeing (Small et al. 2014), adolescent medication adherence and caregiver comfort in communicating with their children about sensitive topics (Bhana et al. 2014).

Despite tentative evidence of the benefits of improved or more frequent communication with others, particularly caregivers, it is rare for adolescents with HIV to talk about the condition (Proulx-Boucher et al. 2011; Rydstrom et al. 2013). Where HIV communication does take place within the family, it is often one-way, focussing mainly on caregivers providing advice or instruction about medication adherence, appointments, protecting others from infection and offering spiritual guidance (Proulx-Boucher et al. 2011; Vaz et al. 2010). Young people have reported questions being ignored or deflected and frequent episodes of being told ‘not to worry.’ (Vaz et al. 2010). Adolescents have also described not wanting to discuss HIV to prevent mothers feeling guilty and of avoiding conversations to prevent other family members from becoming upset (Proulx-Boucher et al. 2011).

Given the potential importance of HIV communication a measure of its determinants would be useful. An existing measure of the frequency and comfort with communication about sensitive issues (including HIV) has been used (Bhana et al. 2016) but there is no measure of *beliefs* about HIV communication. Ideally such a scale would contain items that covered constructs contained in relevant health behaviour theories such as the Theory of Planned Behavior (Ajzen 2011), emphasising the importance of HIV communication attitudes (evaluation of expected outcomes of communication), subjective norms (perceived social pressure to communicate), and perceived behavioural control (subjective sense of control over communication). The development of a brief, reliable measure of HIV communication beliefs for adolescents with HIV may help in the development or assessment of interventions that aim to enhance HIV communication and social support.

This study aimed to develop a brief measure of HIV communication beliefs for adolescents with PAH, aged 12 to 16 years of age. This age range is one where those living with PAH will be likely to know they have HIV (WHO 2011) and may have shared their status with others. As this developmental stage is one where sexual onset may not have occurred for some, we sought to construct a measure that assessed HIV communication thoughts and feelings to family, friends *and* partners. We hypothesised that higher scores on a reliable measure of HIV communication beliefs (e.g., more positive HIV communication attitudes) would be related to higher rates of HIV communication intention and frequency.

## Method

### Participants

Sixty six adolescents with HIV participated out of 67 who had consented to complete measures as part of their attendance at a UK residential intervention (a week-long intensive peer engagement support camp). The intervention was offered to all 12–16 year olds in the UK with HIV. Seventy seven young people attended the intervention. Demographic and clinical characteristics, obtained from participants and the Collaborative HIV Paediatric Study (CHIPS) database (Judd et al. 2007), appear in Table 1.

**Table 1:** Sample demographic and clinical characteristics (n = 66)

Gender	Frequency (%)
Female	39 (59)
Male	27 (41)
Age (in years)	
12	13 (20)
13	6 (9)
14	13 (20)
15	20 (30)
16	14 (21)
Region of birth	
Africa	34 (52)
UK	27 (41)
Other Europe	2 (3)
Asia	2 (3)
Not specified	1 (2)
Age at Naming/	
10	17 (26)
Paediatric disclosure (in years)	
10–12	38 (58)
>12	8 (12)
Not specified	3 (5)
Ethnicity (n = 61)	
Black African	45 (74)
Mixed	9 (15)
White	5 (8)
Other	2 (3)
CD4 count (mm^3^, n = 59)	
Median	707
IQR	513–942
Viral Load (copies/mL, n = 43)	
<50	43 (81)
>50	10 (19)
Antiretroviral regimen (n = 55)	
Nucleoside reverse transcriptase inhibitors (NRTIs) + protease inhibitor	23 (42)
2NRTIs + Nevirapine	15 (27)
2NRTIs + Efavirenz	13 (24)
Other	4 (7)

### Procedure

The *item generation phase* involved the author (a clinical psychologist with extensive HIV clinical and research experience) and a senior member of the UK Children’s HIV Association’s (CHIVA) staff (with extensive experience as a social worker and provider of services to young people living with HIV) reviewing the literature and generating questions using the Theory of Planned Behavior (Ajzen 2011) as a guide. A list of potential items was developed. In the *piloting phase*, members of the CHIVA Youth Committee (CYC) (young people with HIV) were asked to suggest additional items and areas for questions and to state whether draft questions were clear, understandable, and relevant. The list of items was redrafted based on this feedback (e.g., simplifying wording and adding the word ‘not’ in bold for one item). The *administration phase* took place at the start of the intervention (August 2015) before any intervention activities. Parental consent was obtained prior to written informed consent/assent from adolescents. The draft measure was administered in paper and pencil form, face to face. The *reliability phase* involved the use of Principal Components Analysis and examination of internal consistency.

The *validation phase* involved exploring the relationship between the measure and other variables (all asked at the same time as the draft measure was administered, except for HIV communication occurrence and frequency at 6 month follow-up in February 2016).

### Measures

HIV communication occurrence at baseline and 6 month follow-up, “*In the last 6 months, have you spoken to anyone about your HIV (not part of your clinic or working for an HIV organisation)?* Pro HIV communication beliefs were expected to be related to HIV communication occurrence at both time points.

HIV communication frequency at baseline and 6 month follow-up, *“How often do you talk about HIV with someone who is not at the clinic or working for an HIV organisation?”*, with responses on a 5 point likert scale from *never* to *daily*. Pro HIV communication beliefs were expected be related to HIV communication frequency at both time points.

HIV communication intention, “*I intend to talk to people more about my HIV in the next 6 months.”* Responses were on a 5 point likert scale*: strongly disagree* to *strongly agree*. Pro HIV communication beliefs were expected be related to higher levels of HIV communication intention.

HIV disclosure cognitions and affect. *The Adolescent HIV Disclosure Cognition and Affect Scale* (Evangeli 2017) was used. This 18-item measure includes items such as *“I am confident that I can make the right choices about whom to share my HIV status.”* Response were on a 5 point likert scale from *strongly disagree* to strongly agree (α = 0.80). Pro HIV communication beliefs were expected be related to higher levels of pro HIV disclosure cognition and affect.

HIV stigma. Seven items from the *HIV stigma scale for children (HSSC-12)* (Wiklander et al. 2013) were used, assessing HIV disclosure concerns, concerns with public attitudes about HIV and personalized stigma. An example item was, “*I have lost friends by telling them I have HIV*” (α = 0.72). Responses were on a 4-point likert scale from “*strongly disagree*” to “*strongly agree*”. HIV communication beliefs were expected to be related to lower levels of HIV stigma.

Quality of life. The *KIDSCREEN-10* index *(*Ravens-Sieberer et al. 2007) was used. This assesses physical and psychological well-being, autonomy and parent relation, social support and school environment within the last week. An example item *was, “Thinking about the last week…Have you felt sad?”* Responses were on a 5-point likert scale from “*never*” to “*always*” (α = 0.83). Pro HIV communication beliefs were expected to be related to higher levels of quality of life.

Self-perception. The 5 item self-perception subscale from KIDSCREEN—52 was administered (Ravens-Sieberer et al. 2005). An example item was, “*Have you been happy with the way you are?*” Responses were on a 5-point likert scale from “*never*” to “*always*” (α = 0.74). Pro HIV communication beliefs were expected to be related to higher levels of self-perception.

### Data Analyses

Analysis used SPSS 21. The distribution of missing data was assessed with Little’s Missing Completely at Random Test (Little 1988) before using Expectation Maximisation to impute missing data. Principal Components Analysis (PCA) was then carried out using both orthogonal (varimax) and oblique (oblimin) rotations. Scree plots were examined to determine the number of factors to extract and analyses were re-run specifying the number of factors. Items were dropped from the scale if factor loadings were low or if they loaded on more than one factor. If any items were dropped, PCA was re-run. Cronbach’s alpha was calculated for the final total scale and its subscales, and item-total correlations were examined. The relationship between the measure and other variables was assessed using independent t tests and Pearson’s correlations, with bootstrapped confidence intervals where parametric assumptions were not met. Two tailed tests were used.

## Results

### Item Generation, Piloting and Administration

The item generation phase produced ten items (six attitude, one normative belief and three self-efficacy). Feedback during piloting resulted in two attitude items being dropped, one normative belief item added, and wording simplified. For each item on the resulting nine-item measure (four attitudes, two normative belief and three self-efficacy items), participants were asked. “*How much do you agree with the following statement on talking about your HIVwith people who know that you are HIV positive?”*, with responses on a five-point likert scale from *strongly disagree* to *strongly agree*. Item scores were added to produce a total score. Higher total scores indicated more pro HIV communication beliefs.

### Principal Component Analysis and Reliability

There was minimal missing data (1.3%), which was missing at random. Thus, Elaboration Maximisation was used to impute data. PCA was then carried out. PCA was used instead of Exploratory Factor Analysis as the measure was novel, with no existing empirical theory about the structure of relationships between items (Brown 1999).

**Fig. 1:**
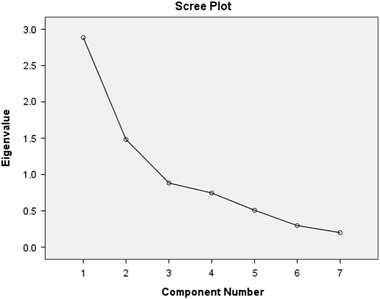
Scree plot for The Adolescent HIV Communication Belief Scale

Examining correlations between the items resulted in one item being dropped due to low correlations with other items. Extraction based on the both Kaiser criteria and the point of inflexion in the scree plot suggested a two factor solution (see Fig. 1 for final scree plot).

Following PCA, one item was dropped due to loading on more than one factor. The final seven-item measure (minimum score 7, maximum score 35) consisted of four items loading on the first factor, and three items loading on the second factor. The combined variance explained by the two factors was 62.4%. The Kaiser-Meyer-Olkin Measure of Sampling Adequacy (0.662) and Barlett’s Test of Sphericity (p < 0.001) were both acceptable (Field 2013). Both orthogonal (varimax) and oblique (oblimin) produced the same factor solution. The measure had a Flesch-Kincaid reading ease score of 87 (understood by an average 11 year-old student). Table 2 shows the factor loadings for all items (those >0.4 displayed).

**Table 2:** Summary of principal components analysis for the adolescent HIV communication belief scale

Factor	Item	Mean (SD)	Factor loading	Eigenvalue	Variance %
1	I am confident that I can get the right support that I need if I talk to people about my HIV	3.86 (1.08)	0.79	2.89	41.23
	I am confident that I can choose the right time and place to talk to others about my HIV	3.74 (1.06)	0.78		
	I am confident that I can talk to people about my HIV if I need to	3.50 (1.15)	0.67		
	Most people who are close to me think I should talk more about HIV with others	2.94 (1.17)	0.65		
2	It makes me feel better	3.79 (0.95)	0.89	1.48	21.17
	It makes it easier to take my medication and look after my health	3.87 (1.01)	0.80		
	It is not helpful^a^	3.93 (0.99)	0.77		

^a^Reverse scored

Cronbach’s alpha for the overall measure was good (α = 0.74), with subscale alphas both good (factor 1, α = 0.71; factor 2, α = 0.78). Females scored higher than males on the overall measure (M 27.21, SD 4.14 versus M 23.36, SD 4.45) (t(64) = 3.60; p = 0.001, d = 0.90) – a large effect size. Females also scored higher than males on subscale one (M 14.87, SD 3.03 versus M 12.85, SD = 3.27) (t(64) = 2.59 p = 0.01, d = 0.85) – a medium to large effect size, and subscale two (M 12.34, SD 1.91 versus M 10.52, SD = 2.78) (t(42.60) = 2.96; p = 0.005, d = 0.79) – a large effect size. Age was not related to the measure or its subscales. Whether participants were born in the UK or not was not related to the measure or its subscales.

### Factor Interpretation

Factor 1 was labelled *communication self-efficacy and normative beliefs*. Example items included, “*I am confident that I can talk to people about my HIV if I need to”*. Factor 2 was labelled *communication attitudes*. Example items included, *“It makes it easier to take my medication and look after my health”*.

### Validity

#### Criterion-related validity

Participants who had communicated about HIV to anyone in the previous 6 months at baseline had a higher total score (M 26.57, SD 3.76) than those who had not (M 24.91, SD 5.17). This difference was not statistically significant (t(64) = 1.46; p = 0.15, d = 0.37), reflecting a small to medium sized effect. There were no significant differences between HIV communication and subscale scores, although the difference was close to significant for subscale one (communication: M 14.86, SD 2.63 vs no communication: M13.40, SD 3.58, p = 0.07, d = 0.46)—a medium effect size. There were no significant relationships between the measure and HIV communication occurrence at follow-up, and no significant relationships between HIV communication frequency in the previous 6 months at both time points, and either total or subscale scores.

There was a positive significant relationship between total score on the measure and HIV communication intention at baseline (r (64) = 0.48, p < 0.01). There was also a positive significant relationship between HIV communication intention and subscale one (r (64) = 0.55, p < 0.01) but no relationship with subscale two.

#### Construct validity

There was were positive relationships between HIV disclosure affect and cognitions and both total score (r(62) = 0.29, p = 0.02) and subscale one (r (62) = 0.29, p = 0.02) of the measure but no relationship with subscale two. The relationships between higher HIV stigma and both total score (r (60) = 0.41, p = 0.001) and subscale two (r (60) = 0.45, p < 0.001) of the measure were significant, but there was no relationship with subscale one. There were no significant relationships between the measure (total or subscales) and either quality of life (e.g. r(56) = −0.01, p = 0.93 for total score) or self-perception (e.g., r(57) = −0.07, p = 0.58 for total score).

## Discussion

This study developed a reliable measure of HIV communication beliefs for adolescents with PAH. Two factors were extracted, representing communication attitudes and normative beliefs, and communication self-efficacy. The separation of attitudes and self-efficacy is consistent with health behaviour models applied to HIV-related behaviours (Ajzen 2011; Fisher et al. 2006). The inclusion of normative belief and attitude items within the same subscale suggests that a clear separation between communication attitudes and normative beliefs may not be present in this population, contrary to the Theory of Planned Behavior (Ajzen 2011).

The expected relationships between the measure and other variables were sometimes, but not always, observed. The effect size of some relationships (e.g., between the total and subscale one scores and HIV communication occurrence in the last 6 months) suggests that this may be due to the study being underpowered. Significant relationships were, however, found between the total score (and subscale one) and both HIV communication intention and HIV disclosure cognition and affect. These relationships suggest that beliefs and feelings about HIV disclosure, HIV communication beliefs and intending to communicate about HIV may be related to each other in a meaningful way. A larger longitudinal study, perhaps in the context of an intervention aiming to enhance onward disclosure and HIV communication may help to explore these relationships.

The relationship between the overall measure and HIV stigma was unexpected. More positive communication beliefs were related to higher HIV stigma. Many of the stigma items were dependent on having disclosed one’s HIV status. Therefore, it may have been those participants who had disclosed were those that remained more positive about both disclosure and subsequent HIV communication than others, despite experiencing some negative disclosure consequences. The relationships between both self-perception and quality of life, and the measure, were weak, perhaps calling into question whether these were appropriate constructs for the assessment of validity. There have been inconsistent relationships between communication and wellbeing in another study (Bhana et al. 2014). It may be that the other variables assessed (communication intention, disclosure attitudes and cognition) are more closely related to HIV communication beliefs.

Given the absence of a multi-item, multi-dimensional scale of HIV communication (in any HIV-positive population), the measure is an important addition to the literature. It is brief, at an appropriate level of complexity for the target population, reliable, and potentially adaptable to other HIV-positive populations. In relation to the sample size it has been shown that if a factor has four or more loadings greater than 0.6, it is reliable regardless of sample size (Guadagnoli and Velicer 1988). The current scale met this criterion for subscale 1. In addition, the fact that the mean communalities were above 0.6 in this study may justify a sample size of less than 100 (MacCallum et al. 1999). Finally, the Kaiser-Mayer-Olkin measure of sampling adequacy was within the acceptable range (Field 2013), and the significant relationships between the measure and communication intentions and disclosure affect and cognitions, suggests that the sample size was satisfactory for the majority of study aims.

### Limitations and Future Research Directions

In relation to limitations, it is not possible to determine whether communication beliefs for adolescents with PAH attending this residential intervention differ from other population in either high or lower income contexts. Demographic and clinical characteristics were, however, consistent with national data (CHIPS 2015). Some aspects were not assessed (e.g., preparedness to communicate and satisfaction with current level of communication). Finally, the measure assessed communication beliefs in relation to *any* recipient. This produced a more generally applicable scale that can be administered to adolescents with HIV regardless of their relationship status. Adolescents with HIV may, however, hold different communication beliefs dependent on the recipient. This possibility could be tested by adapting the wording of the measure for specific recipients, although it will be important to assess the reliability and validity of the scale under these altered conditions.

Further work in developing the measure could involve larger samples and assessing its relationship with internalised HIV stigma. The measure may need to be adapted for use with related populations (e.g., behaviourally infected adolescents) and both translated and culturally adapted for use in regions with a greater prevalence of adolescents with PAH. Validity could be established more comprehensively for the measure as a whole and its subscales. The development of relevant illness communication theory could assist in the selection of appropriate constructs for testing the measure’s validity.

The existence of an HIV communication scale may allow for theoretical models of HIV communication to be developed. It may also be clinically useful when an in-depth assessment of HIV communication determinants is required as part of therapeutic work with adolescents with HIV. The measure may also help in the development of testing of future interventions to enhance HIV communication in adolescents living with HIV. For example, the close relationship between the measure and communication intention and disclosure affect and cognitions, suggests that communication self-efficacy, normative beliefs and attitudes could all be part of an intervention to enhance HIV communication.
